# Does a nurse-led postpartum self-care program for first-time mothers in Bangladesh improve postpartum fatigue, depressive mood, and maternal functioning?: a non-synchronized quasi-experimental study

**DOI:** 10.4069/kjwhn.2021.09.08

**Published:** 2021-09-30

**Authors:** Fahima Khatun, Tae Wha Lee, Hye Jung Lee, Jeongok Park, Ju Eun Song, Sue Kim

**Affiliations:** 1 National Institute of Advanced Nursing Education and Research, Dhaka, Bangladesh; 2 College of Nursing, Mo-Im Kim Nursing Research Institute, Yonsei University, Seoul, Korea; 3 College of Nursing, Ajou University, Seoul, Korea

**Keywords:** Fatigue, Intervention, Postpartum depression, Postpartum period, Self-care

## Abstract

**Purpose:**

This study aimed to test the efficacy of a nurse-led postpartum self-care (NLPPSC) intervention at reducing postpartum fatigue (PPF) and depressive mood and promoting maternal functioning among first-time mothers in Bangladesh.

**Methods:**

A non-synchronized quasi-experimental design was used. First-time mothers were recruited during postpartum and assigned to the experimental or control group (34 each). The experimental group received the NLPPSC in the hospital, a 1-day intervention that focused on increasing self-efficacy. The control group received usual care. Data on PPF, depressive mood, maternal functioning, self-care behaviors, postpartum self-efficacy, and self-care knowledge were collected at postpartum 2 weeks (attrition 23.5%) and 6 weeks (attrition 16.1%). Data were analyzed using descriptive statistics, bivariate statistics, and linear mixed model analysis.

**Results:**

One-third (33.3%) of new mothers experienced depressive mood (Edinburgh Postnatal Depression Scale scores of ≥13 points). The NLPPSC intervention was statistically significant in decreasing PPF (β=–6.17, SE=1.81, t=–3.39, *p*<.01) and increased maternal functioning at postpartum 6 weeks in the experimental group (β=13.72, t=3.73, *p*<.01) compared to the control. Knowledge was also statistically significant for increased maternal functioning over time (β=.37, SE=.18, t=2.03, *p*<.05). However, there were no statistically significant differences in depressive mood over time.

**Conclusion:**

The NLPPSC intervention was feasible and effective in improving fatigue and maternal functioning in Bangladeshi mothers by postpartum 6 weeks. Postpartum care knowledge was effective in improved maternal functioning and thus supports implementing the NLPPSC intervention for new mothers after childbirth.

## Introduction

Postpartum fatigue (PPF) has a negative impact on maternal depression [1] and self-care in daily functioning [2,3]. Inversely, maternal functioning has a significant positive correlation with first-time mothers’ self-efficacy (SE) [4]. Thus, less PPF can be influential in increasing maternal functioning. Nurses are in a key position to intervene to alleviate PPF [5], screen for depression [6], and increase maternal functioning [7] for the well-being of mothers and newborns in the early postpartum period. However, according to the 2014 Bangladesh Demographic and Health Survey [8], 63.6% of mothers do not receive care from trained personnel within 2 days of childbirth in Bangladesh. This is most likely due to the high rate of home births (69%), which is reflected in Bangladesh Bureau of Statistics data [9].

The findings of a quasi-experimental study on safe motherhood [10] highlighted an urgent need for programs in Bangladesh to increase knowledge and SE, and positively affecting maternal health outcomes. Complications such as PPF, depressive mood, and maternal malfunctioning are important issues that require special attention from nurses and health care professionals, especially for new mothers. Also, a an earlier randomized control trial (RCT) [11] reported that compared to providing standard care, it was more feasible and cost-effective to implement a nurse-led intervention for care. In order to promote maternal health in Bangladesh, some communities and rural areas are covered by programs such as the International Center for Diarrheal Disease and Research, Bangladesh, and the Bangladesh Rural Advanced Community, which provide door-to-door services including identifying complications and arranging referrals [12]. In addition, the government is expanding health access through electronic media, especially for maternal health services. For example, national electronic health (eHealth) strategies have been initiated to incorporate information communication technologies into the Bangladesh health system to align with the Digital Bangladesh Vision 2021 [13]. Various stakeholders are involved, especially the Mobile Alliance of Maternal Action Bangladesh [14], which is a leading mobile health service for maternal health outcomes. However, these services lack opportunities for exchange and assurance of understanding, or individualized care, and cannot substitute care provided by health care professionals in the early period.

In Bangladesh, these findings underscore the need for particular attention on PPF, including access to care and increased awareness and knowledge of activities, especially among young mothers who lack resources and those with vulnerabilities such as fatigue or depression. Maternity nurses' initiation of PPF management has been shown to increase efficacy [3], and nurses have a role to promote mothers’ knowledge and influencing mothers to undertake at home. Maternal nurses—who are mostly women—also fit the cultural expectations of Bengali women regarding care and play a strategic role in delivering postpartum care. However, there is a current lack of studies focusing on nurse-led programs in Bangladesh.

This study aimed to test the efficacy of a nurse-led postpartum self-care (NLPPSC) program that was developed for first-time mothers in Bangladesh, on maternal health outcomes. The specific goals were as follows:

First, it examined the differences in process indicators (SE, and postpartum care knowledge) within and between the experimental and control groups.

Second, it examined the differences of outcome indicators (PPF, depressive mood, and maternal functioning) between the experimental and control groups, over three time-points. In this study, it was hypothesized that mothers in the experimental group would demonstrate the greater decrease in PPF than the control group, greater decrease in depressive mood than the control group, and greater increase in postpartum maternal functioning than the control group.

## Methods

Ethics statement: This study was approved by the Institutional Review Board of Yonsei University College of Nursing (YUCON-IRB 2017-0021). Informed consent was obtained from the subjects.

**Study design:** This study employed a non-synchronized quasi-experimental design with a baseline and two post-test periods. To avoid diffusion of the intervention, data from the control group were collected first (May to June 2017), followed by the experimental group (July to September 2017). This study report followed the TREND reporting guidelines [15].

**Participants**: Women who delivered and received postpartum care at Dhaka Medical College Hospital (DMCH), Dhaka, Bangladesh, were recruited via convenience sampling. DMCH is a large public tertiary hospital where women from across Bangladesh come to give birth.

***Inclusion criteria were*** (1) First-time mother, (2) spontaneous vaginal delivery, (3) newborn 5 min APGAR ≥ 6, (4) postpartum duration ≤1 week; and (5) ability to read and understand written Bengali. ***Exclusion criteria were*** (1) multiple infants, (2) chronic disease (i.e., diabetes, hypertension, bronchial asthma, etc.) or any postpartum complication (i.e., postpartum bleeding, puerperal sepsis, or psychosis), (3) had received newborn’s vaccines at places other than DMCH and the Expanded Program of Immunization (EPI) center in Mohakhali, Dhaka, and (4) no access to telephone. For recruitment, flyers were posted around the postpartum unit to invite new mothers to voluntarily join the study under the permission from hospital executives. Potential participants could show their interest by approaching nurses on the postpartum ward, who acted as a ‘bridge’ between the potential participants and the researcher. The ward-in-charge nurse advertised the study and performed the initial screening for potential participants.

**Figure 1.**
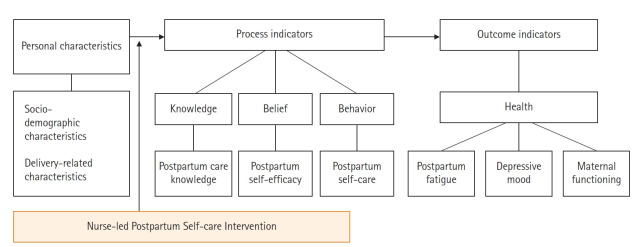
Conceptual framework of the study.

**Interventions**: The conceptual framework for this study was based on the concepts of SE as a component of social cognitive theory [16] and linked to health as the final outcome. postpartum SE has been shown to positively affect mothers’ ability to perform self-care behaviors and promote postpartum health [17,18]. A modified conceptual framework for postpartum SE was conceptualized for this study in three constructs (Figure 1): (1) personal characteristics (sociodemographic and delivery-related characteristics) was postulated to influence (2) process indicators (postpartum care knowledge, postpartum SE, and postpartum self-care); and (3) outcome indicators (PPF, depressive mood, and maternal functioning). The NLPPSC program was posited to affect process and outcome variables. The NLPPSC program built on the four sources of SE, enactive mastery, vicarious experience, verbal persuasion, and physiologic arousal, and ultimately aimed to alleviate PPF and depressive mood, and positively affect maternal functioning.

Sample size: It was calculated by using G*Power, with effect size dz 0.5; α of 0.05; and Power (1-β error probability) 0.95, which showed a minimum of 27 participants in each group. The expected attrition rate was estimated at range 15%-25% due to possibility of drop out in 2 and 6 weeks post intervention [3]. Therefore, 68 participants were recruited allocated to the experimental (n=34) or control groups (n=34).

### Instruments

The questionnaire set consisted of 104 items, which took around 20-30 minutes to answer. Permission to use instruments was obtained from the original authors unless available for free access. For instruments not available in Bengali, translation-back translation was done and independently reviewed by Bangladesh experts (English lecturer and obstetrician) to check for appropriate meaning and to ensure the equivalence of the two versions.

### Outcome indicators

#### Postpartum fatigue

The 10-item Postpartum Fatigue Scale [19] measured on a 4 point Likert scale (1= not at all; 4=all the time) was used. It consists of physical fatigue (four items) and mental fatigue (six items) dimensions. Total summed scores can range from 10-40, and higher scores indicate greater fatigue. Internal consistency was .77 in the original study [19] and in this study the Cronbach’s alpha ranged from .35 to .82 across the three time points.

#### Depressive mood

Depressive mood was measured by using the 10-item Edinburgh Postnatal Depression Scale [20]. A 4-point Likert (0-3) is used and higher summed scores possible (range, 0-30) indicate greater depressive symptoms. In Western countries a cutoff of greater than 12 or 13 indicates the need for professional aid [20]. Due to cultural stigmatization towards postpartum depression in Bangladesh [21] and noting prior research [3], 13 or above was used as the cutoff for this study. Cronbach’s alpha was .86 in the original study [20], .70 in a prior survey of Bangladesh new mothers [22], and ranged from .48 to .56 across the three time points in this study.

#### Maternal functioning

Postpartum maternal functioning was measured using the Barkin Index of Maternal Functioning (BIMF) [23]. The BIMF is a 20 item 7 point Likert scale (0 strongly disagree, 6 strongly agree) and scores are summed (possible range, 0-120), with higher scores indicating higher postpartum maternal functioning. The Cronbach’s alpha of this tool was .87 at development [23] and ranged from .53 to .89 across the three time points in this study.

### Process indicators

#### Postpartum self-care

Denyes’(1990) 8-item Self-Care Practice Instrument (DSCPI-90^©^) [24] was used as a general measure of self-care actions that meet universal self-care requisites, and items were adapted for the postpartum context. A 7 point Likert (1=strongly disagree, 7=strongly agree) is used and items are summed with a total possible range of 18-126 points. Higher scores reflect higher levels of self-care behavior. Cronbach’s alpha coefficients have been reported as between .82 to .89 [24] and ranged from .47 to .86 across the three time points in this study.

#### Postpartum self-efficacy

Postpartum SE was measured by modifying the Postpartum Management Self Efficacy Tool [25]. From the original 16 items, one item (sitz bath use) was deleted, as it was not relevant for use in Bangladesh context. The 15 items were scored on a 4 point Likert scale (1=not confident at all, to 4=very confident) and higher summed scores indicated higher levels of postpartum SE (possible range,15-60). Cronbach’s alpha was .90 at development [25] and ranged from .33 to .93 across the three time points in this study.

#### Postpartum Care Knowledge

The 20-item Postpartum Self-Care Knowledge scale [26] was modified, by deleting one item on sitz bath knowledge that was not relevant to Bangladesh context. Only correct answers received a score and scores for the 19 items were added to obtain a total score (possible score range, 0-19). Higher scores indicated high level of postpartum care knowledge. The Kuder Richardson-20 of this instrument in the original study was .76 at baseline and .73 for posttest [26] and for this study ranged from .55 to .85 across the three time points.

#### Personal characteristics

Personal characteristics consisted of socio-demographic characteristics (five items) and delivery-related characteristics (seven items), derived from the literature.

### Procedures

The data were collected by the first author with help from two trained local research assistants (RA), from May to December, 2017. The data were collected at baseline, postpartum 2 weeks, and postpartum 6 weeks.

***1) Baseline*** (between postpartum day 1-6): Participants returned completed questionnaires in a collection box placed in the ward and the researcher checked for any missing items. Phone number were obtained for the purpose of further contact. The posttest 1 questionnaire was provided along with a stamped envelope and participants were requested to complete the second questionnaire and send it via mail 2 weeks later. The researcher provided a card marked with the code number to show the RA at posttest to avoid unnecessary exposure of personal information.

The NLPPSC was developed following the ADDIE model [27], according to the following phases (Supplementary material).

***Analysis phase:*** Data on postpartum care and interventions from WHO, UNFPA, World Bank, and a literature review was done using PubMed, CINHAL, EMBASE, Cochrane, PsycInfo, and Google Scholars database, for English publications from 2000-2017. Eleven related articles were identified but none were found for Bangladesh. Interviews with three first-time postpartum mothers in Bangladesh, who were purposively selected, explored actual postpartum needs and wishes for aid from maternal nurses or midwives, especially relating to practicing self-care behaviors at home. Participants noted at least one home visit and/or telephone calls could bridge the connection with maternal nurse/midwives and mothers and would be helpful to carry out effective postpartum self-care. ***Design phase:*** Program components built on using SE sources [28] of verbal persuasion (face-to-face discussion with interpretation of the participants’ responses, supplemented by a brochure containing postpartum care information). SE enactive mastery and vicarious experiences were designed as providing opportunity for participants to demonstrate skills and role play how to perform. The intervention period was drafted to be provided in the hospital before discharge, as two 30-minute sessions to accommodate maternal attention span to avoid fatigue. The initial program consisted of two phone calls at 2nd and 4th weeks to stimulate mothers to perform self-care actions and to clarify any ambiguities. ***Development phase:*** Program content and format were reviewed by an expert panel in Bangladesh (n=5) consisting of 2 obstetricians, 1 head nurse of the postpartum unit, and 2 women’s health nursing faculty. Panel consensus were obtained with criteria of CVI >85% for each item (mean CVI was .94). Timing for the second follow-up was modified to occur at postpartum 6 weeks. *Implementation & evaluation phase:* The program was provided to 2 postpartum mothers by convenience sampling at DMCH, using the same inclusion/exclusion criteria for the main study. Based on participant suggestions, we determined a larger space was necessary for the small groups and decided to add using a model placenta and uterus and a newborn doll. Thus, the NLPPSC program was finalized.

The experimental group received the finalized NLPPSC (Table 1), face-to-face education in four 30-minute sessions (small group format of 3-5 mothers) over 2 days during hospital stay, and reinforcement by phone calls (duration 3-5 minutes each) at postpartum 2 weeks and 4 weeks. The control group received the hospital’s standard care (5-minute verbal instruction at the time of hospital discharge).

***2) Posttest 1*** (postpartum 2 weeks): The response rate for the mailed questionnaires was 76.4%.

***3) Posttest 2*** (postpartum 6 weeks): Mothers were contacted to identify date and location of the EPI center they would visit for infant vaccination. A trained research assistant met the participant and checked the card’s code number, provided the questionnaire, which was completed on site. Upon completion a gift voucher of 320 Bangladeshi taka (BDT; approximately 4 US dollars) was given as a token of appreciation for participation. For the control group, a postpartum self-care brochure was provided at the end of data collection along with gift voucher. The response rate of participants was 83.8%.

### Data analysis

The data were analyzed by IBM SPSS statistics version 21 (SPSS Inc., Chicago, IL, USA). Descriptive statistics, bivariate statistics t-test, chi-square was used to examine homogeneity of variables, and paired sample t-test, and independent sample t-test were used to examine differences within and between groups. Due to attrition rate in follow up (23.5% at 2 weeks, 16.1% at 6 weeks) linear mixed model (LMM) was used to adjust for covariates and examine the treatment effects over three time periods. LMM was used for repeated measurements to explore the change in PPF, depressive mood, and maternal functioning between baseline and at 2 weeks; and baseline and 6 weeks while adjusting age, living status, and family income for baseline differences in the two groups. The LMM is a powerful advanced statistics tool that can estimate changes over time despite missing data points of a subsample, and thus, data from all 68 participants were included for analysis.

## Results

**Figure 2.**
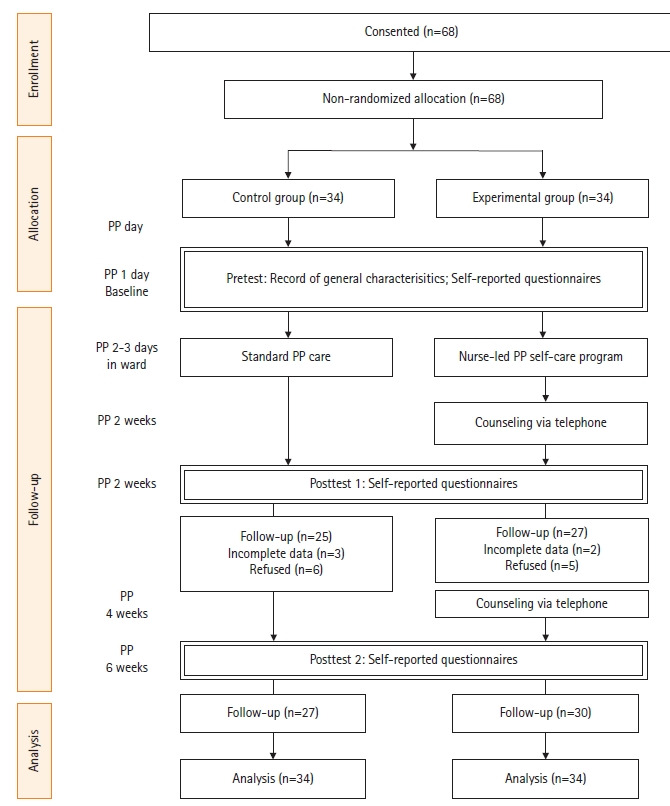
Flowchart of participants. PP: postpartum.

**Participation flow**: Flow of participation through each stage is presented in Figure 2.

**Recruitment**: Data from the control group were collected from May to June 2017, followed by the experimental group from July to September 2017.

### Baseline data

The average age of the participants was 20.71 years old. Most lived with their husbands without extra family members (61.8%), and did not work out of the home (85.3%). Monthly family income averaged 25,155 BDT (approximately 300 US dollars). The average gestational age at delivery was 37 weeks, and newborn conditions were generally good (APGAR scores of 8 and more, 92.6%). Forty-seven percent had participated in at least one parenting preparation class, and all had at least one antenatal care (ANC) visit, of whom 52.9% had received at least three ANC, the minimum number recommended by WHO.

The experimental group was slightly older (t=2.90; *p*<.01), and more participants tended to live with their husband, including other family members (χ^2^=3.98; *p*<.05). They also showed slightly higher income compared to the control group (t=-2.38; *p*<.01). There were no differences in delivery-related information or main variables between the experimental and control groups (Table 2).

### Baseline equivalence

For baseline PPF, both the experiment (mean±SD, 26.32±3.21) and control groups (24.68±3.18) showed a moderate level and the experiment group showed statistically significantly higher fatigue (t=-2.17, *p*<.05). Depressive mood (cutoff of ≥13) was noted in both the experimental (33.3%) and control group (29.6%), although there were no statistically significant differences (χ^2^=-.29; *p*=.76). The level of postpartum maternal functioning was slightly less than midpoint, and postpartum maternal functioning was not significantly different between the groups.

### Differences from baseline to 2 weeks

**Figure 3.**
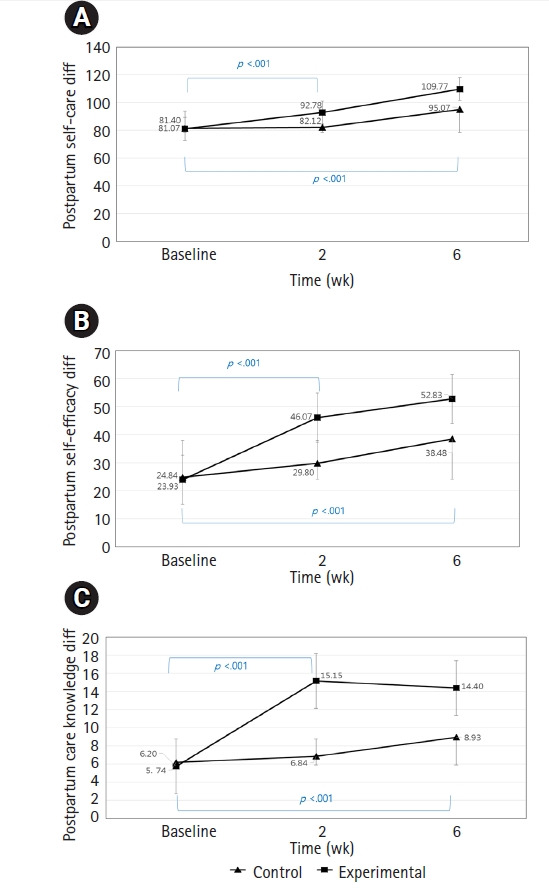
Differences (diff) in postpartum self-care (A), self-efficacy (B), and self-care knowledge (C) between the two groups over time.

The process variables, postpartum self-care, postpartum SE, and postpartum care knowledge are conceptually meaningful for effects on the output variables. Within the experimental group between baseline and postpartum 2 weeks, all variables showed significant improvement, while for the control group, only postpartum SE was improved (t=-9.08, *p*<.01). For between group differences at postpartum 2 weeks, postpartum self-care (M_dif_ 10.98; t=9.49, *p*<.01), postpartum SE (M_dif_ 17.18; t=18.30, *p*<.01) and postpartum self-care knowledge (M_dif_ 8.76; t=11.08, *p*<.01) of the experiment group were significantly higher compared to the control group (Figure 3).

### Differences from baseline to 6 weeks

From baseline to postpartum 6 weeks, the experimental group showed significantly greater differences (*p*<.01) compared to the control group (Figure 3). For within group differences, there were significant mean differences in all three variables for both the experimental and control groups: For example, the experimental group’s postpartum self-care (M_dif_ 15.28; t=10.02, *p*<.01), postpartum SE (M_dif_ 15.04; t=19.72, *p*<.01) and postpartum care knowledge (M_dif_ 5.82; t=6.91, *p*<.01) levels all improved by 6 weeks. The same pattern was found for the control group, which may suggest that by 6 weeks after childbirth, a natural improvement is likely for mothers in general.

### Effectiveness of the intervention over time

For the LMM analysis to test the effectiveness of NLPPSC intervention on PPF, depressive mood, and postpartum maternal functioning, the covariates that showed statistical significance, i.e., maternal age, living status, and monthly income, were adjusted in the equation. In addition, conceptually meaningful variables of this study, i.e., postpartum self-care behavior, postpartum SE, and postpartum self-care knowledge, were also loaded as covariates (Table 3).

**Effects on PPF**: The mothers who received the NLPPSC intervention showed statistically significant levels of slightly higher fatigue (β= 1.45, SE=.72, t=1.98, *p*<.05) over time when compared to the control. However, the treatment effects of NLPPSC intervention group at 6 weeks postpartum was statistically significant in decreased fatigue (ß= -6.17, SE=1.81, t= -3.39, *p*<.01) compared to the control group.

**Effects on depressive mood:** The estimated treatment effects did not find significant changes related to the NLPPSC intervention for depressive mood compared to the referent group.

**Effects on postpartum maternal functioning**: For postpartum maternal functioning, the estimated treatment effects showed that living with husband but not with other family members, was statistically significant (ß= 4.44, SE=1.30, t=3.39, *p*<.01) compared to women who also lived with other family members. Among other variables, more postpartum self-care knowledge was also statistically significant (ß= .37, SE=.18; t=2.03, *p*<.05) for maternal functioning for the NLPPSC participants. Maternal functioning at both 2 weeks (ß= 12.76, SE=1.55, t=8.20, *p*<.01) and 6 weeks (ß= 24.54, SE=3.12, t=7.84, *p*<.01) showed statistically significant improvement over time. Finally, the treatment effects at 6 weeks were statistically significant (ß= 13.72, SE=3.67, t=3.73, *p*<.01) in increased maternal functioning in the experimental group compared to the control group.

**Ancillary analyses**: There was no ancillary analysis.

## Discussion

### Effectiveness of the NLPPSC intervention

The NLPPSC significantly increased scores in process indicators, postpartum care knowledge, postpartum SE, and postpartum self-care behavior; and postpartum fatigue and maternal functioning was improved among the outcome indicators. As there were no intervention studies found in Bangladesh to directly compare with this study, studies from others countries were compared in the following discussion. Discussion on outcome indicators are presented first, as they were the main study hypotheses.

### NLPPSC effects on postpartum fatigue

Fatigue decreased over time in the experimental group compared to the control. Among the time points (within 2 days after delivery, postpartum 2 weeks, and postpartum 6 weeks), the decrease in fatigue at postpartum 6 weeks was statistically significant in the experimental group. This finding is similar with previous studies that used a psycho-educational program with telephone support to manage PPF with a RCT design [2,3]. Both studies encouraged mothers to prioritize their tasks, and plan for and engage in postpartum self-care behaviors. Although this study found statistically significant improvement in SE at postpartum 2 weeks and postpartum 6 weeks in the NLPPSC group, LMM analysis did not find direct influences of SE for fatigue. This suggests indirect effects of SE on fatigue, which may need to be further explored in future studies. This study found a mid-level of fatigue at baseline. Although using a different measurement of fatigue, a prior survey of new mothers in Bangladesh reported a lower mean fatigue score [22]. This may be related to measurement time or differences in sample characteristics, but further studies are needed to clarify.

### NLPPSC effects on depressive mood

The NLPPSC intervention was not statistically significant in decreasing depression scores, when treated as a continuous variable. This finding is similar to a RCT study of a nurse-led postpartum discharge education program to reduce postpartum depressive mood, which did not find differences at 6 weeks nor at 3 months [29]. In contrast, Giallo’s study (2014) [3] used a fatigue management booklet, a home visit, and three telephone support sessions led by a professional, and was effective in decreasing postpartum depressive mood after 6 weeks. Differences might be due to the intensity and number of contacts, as the NLPPSC did not include home visits and had one less telephone contact. Another RCT with first-time mothers [30] reported that the treatment group (12.5%) had lower depression than the control group (25%) at 6 months postpartum. This suggests the possibility that NLPPSC participants may have shown effects over a longer period of time, assuming continued practice of self-care.

This study found a high proportion of mothers with postpartum depression (33% in the intervention group) at 2-3 days within delivery. Of these mothers, 82.4% had received an episiotomy, which may be related to their perception of fatigue. At 6 weeks, the control group showed a high proportion of postpartum depression (n=9, 33% with scores ≥ 13), much higher than 9% reported in a previous study of Bangladeshi women at 6-8 weeks postpartum [31]. This may be due to demographic differences in the two studies. The previous study involved 100 multiparous postpartum mothers with mean age 25.5 years, of whom 54% had one child, whereas this study mostly included young first-time mothers, who may have been more sensitive to depressive mood.

When analyzing depression based on cut off categories, the pattern of depression over the three time points (up to 6 weeks) in mothers in the NLPPSC group steadily decreased, resulting in none at ≥13 at 6 weeks, which is in contrast to the pattern of increased depression in the control group over time (33% at ≥13 at 6 weeks). Future studies are needed to employ the NLPPSC program with more contact intensity and may be needed to be followed up for longer periods, to more accurately manage depressive moods of new mothers in Bangladesh.

### NLPPSC effects on maternal functioning

In maternal functioning, maternal age and family income had a statistically significant association with increased maternal functioning. Women living with their husband only, reported significantly higher maternal functioning than women also living with other family members. As it happens that after childbirth in Bangladesh, other relatives will often come to support the new mother or she will stay at her parents’ home for some time to aid postpartum recovery, the postpartum living status may temporarily change. Thus, usual living status may not offer clear understanding of how it may be related to postpartum maternal functioning. The process indicator postpartum self-care knowledge was statistically significantly associated with increasing maternal functioning over time. The intervention was significantly effective in increased maternal functioning at 2 weeks and also at 6 weeks follow-up. These findings are supported by findings from the literature. For example, a postpartum educational intervention for first-time mothers consisting of four sessions and phone calls found improvement in maternal functioning status and self-confidence after 8 weeks [7]. In addition, another study [23] found that postpartum knowledge and efficacy influenced maternal functioning.

### NLPPSC effects on process indicators

For the process indicators, self-care behavior, SE, and postpartum care knowledge showed statistically significant increases at both postpartum 2 weeks and 6 weeks, compared to baseline levels. This finding is similar to studies such as a nurse-led self-care intervention for primiparas after birth [32] and a prenatal and postnatal self-care educational program focused on perineal care, nutrition, and sexual activities [17], which found improvement in postpartum self-care knowledge and self-care efficacy.

Overall, the participants in this study were young age (average 20.71 years) and had completed primary to secondary level education (83.8%), which may have helped them to understand the NLPPSC content. In addition, as the majority of the mothers (85.3%) did not work out of the house they may have had sufficient time to perform self-care behaviors at home.

As such, this NLPPSC intervention offers implications for nursing practice, theory development, and research. In practice, maternal nurse caring for new mothers in Bangladesh should provide psycho-educational support using strategies for fatigue management before hospital discharge. Although brief, the NLPPSC was influential in improving the process indicators and improving fatigue and maternal functioning. This would contribute to postpartum mothers performing self-care activities for their wellbeing and their newborns. Although the NLPPSC was planned as a brief practical intervention, in reality the allocated time for intervention took more than usual as participants required more clarification. Therefore, duration of interventional time may need to be increased for future implementation. In regards to nursing theory, the conceptual framework used in this study offers a framework for generating nursing knowledge theoretically. Finally, this study identifies recommendations for further studies. For example, exploratory studies on SE and postpartum depression, and developing culturally appropriate postpartum self-care instruments for use in Bangladesh would be beneficial.

**Limitations:** This study has some limitations. The low values of internal consistency for some instruments may have been a source of measurement error. This may be related to the fact that although the questionnaire was administered face-to-face to facilitate understanding and completion, the timing (immediately after childbirth) may not have been optimal. For example, ‘I am a good mother’, or ‘I hold my newborn comfortably’ may have been sources of measurement and response bias. Another limitation is that participants were recruited from only one public hospital (DMCH), and convenience sampling might be a threat of selection bias. Furthermore, as this study focused on women delivering at the hospital, it cannot be applied to the sizable proportion of Bangladesh women who deliver at home, and future studies are needed for how to pragmatically and effectively reach this needy population.

In conclusion, the NLPPSC was effective in decreasing PPF and increasing postpartum maternal functioning of first-time mothers in Bangladesh. However, it was not found to be effective in decreasing postpartum depressive mood. The NLPPSC was effective in improving SE, self-care behaviors, and postpartum care knowledge at both 2 weeks and 6 weeks. Postpartum care knowledge was effective in improved maternal functioning, the supports implementing the NLPPSC for new mothers following delivery. Although this study focused on hospital-based births, a relatively small proportion of all births in Bangladesh, this nurse-led program was found to be feasible and can be easily provided for first-time mothers who have hospital births. Further studies are needed to expand the NLPPSC in terms of intensity and/or frequency of contact, as well as consider follow up measurement for a longer period. Further exploratory and interventional studies are also needed on postpartum depression, and development of postpartum self-care instruments based on Bangladesh context.

## Figures and Tables

**Table 1.** The nurse-led postpartum self-care program

Time	Mode (time)	Contents
Postpartum day 2	Session 1 (30 minutes)	Discussion (Supplemented by written brochure)
		• Postpartum self-care: general care, perineal care, care of episiotomy wound
		**• **Postpartum fatigue: how to identify signs and symptoms, sources and consequences
		• Postpartum self-care practice: sleeping better, resting, nutritional intake, exercise, prevention of infection, and seeking support for baby care
	(30 minutes)	Demonstration (video clip)
		• Postpartum vaginal discharge (lochia alba, serosa, and rubra)
		• Correct use of sanitary napkins
		• Involution of the uterus
Postpartum day 3	Session 2 (30 minutes)	Discussion (supplemented by written brochure)
		• Postpartum nutrition
		• Prevention of constipation
		• Newborn care: mother-infant relationship, breastfeeding, immunization
	(30 minutes)	Demonstration (video clip on newborn care)
		• Umbilical cord care
		• Changing diapers
		• Baby bathing
2nd week	Phone call 1 (3–5 minutes)	Ask about self-care and clarify any questions
4th week	Phone call 2 (3–5 minutes)	Ask about self-care and clarify any questions
		Reminder to come for newborn vaccination

PP: Postpartum

**Table 2.** Homogeneity testing of participants (N=68)

Characteristic	Categories	Total (N=68)	Exp (n=34)	Cont (n=34)	t or χ^2^	*p*
Age (year)		20.71±2.56	21.61±3.13	19.82±2.00	2.9	.005
Education	Primary	31 (45.6)	16 (47.1)	15 (44.1)	1.0	.60
	Secondary	26 (38.2)	14 (41.2)	12 (35.3)		
	Higher than secondary	11 (16.2)	4 (11.7)	7 (20.6)		
Living status	With husband only	42 (61.8)	17 (50.0)	25 (73.5)	3.98	.046
	With husband and other family members	26 (38.2)	17 (50.0)	9 (26.5)		
Occupation	Not employed	58 (85.3)	27 (79.4)	31 (91.2)	1.87	.17
	Employed	10 (14.7)	7 (20.6)	3 (8.8)		
Income (BDT 1,000)*		25.15± 8.88	27.73±9.35	22.58±8.41	–2.38	.02
Episiotomy	Yes	56 (82.6)	29 (85.3)	27 (79.4)	.40	.52
	No	12 (17.7)	5 (14.7)	7 (20.6)		
Gestational age (week)		37.16±.98	37.09±.99	37.24±.98	.61	.54
PP duration (day)		1.45±.50	1.47±.50	1.44±.50.81	–24	.81
General condition of newborn (APGAR)	Fair (6-7)	5 (7.4)	2 (5.9)	3 (8.8)	1.49	.47
	Good (8)	33 (48.5)	19 (55.9)	14 (41.2)		
	Very good (>8)	30 (44.1)	13 (38.2)	17 (50.0)		
Sex of newborn	Female	31 (45.6)	15 (44.1)	16 (47.1)	.05	.80
	Male	37 (54.4)	19 (55.9)	18 (52.9)		
Participation in parenting class (number of classes)	None	36 (52.9)	15 (44.1)	21 (61.8)	2.19	.33
	1	29 (42.7)	17 (50.0)	12 (35.3)		
	2	3 (4.4)	2 (5.9)	1 (2.9)		
ANC visits	1–2	6 (8.8)	1 (2.9)	5 (14.7)	3.24	.35
	3	36 (52.9)	20 (58.8)	16 (47.1)		
	4 or more	26 (38.2)	13 (38.2)	13 (38.2)		
	Possible score range					
PP self-care	18–126		80.53±4.48	81.53±6.03	.77	.44
PP self-efficacy	15–60		23.65±2.79	24.50±2.13	1.41	.16
PP care knowledge	0–19		5.41±2.83	6.03±2.64	.93	.35
PP fatigue	10–40		26.32±3.21	24.68±3.18	–2.17	.03
Depressive mood	0–30		11.74±3.44	10.97±3.54	– .90	.37
	≥13		10 (33.3)	8 (29.6)	–.29	.76
Maternal functioning	0–120		54.03±6.08	52.38±7.95	–. 95	.34

Values are presented as mean±standard deviation or number (%). Cont: control group; Exp: experimental group; PP: postpartum.

* Bangladeshi taka (BDT) 1,000 is approximately 12 US dollars.

**Table 3.** Estimated effects of the nurse-led postpartum self-care intervention (N=68)

Parameter	Postpartum fatigue			Depressive mood			Postpartum maternal functioning		
	Estimate	SE	t	Estimate	SE	t	Estimate	SE	t
Intercept	22.71	5.21	4.36***	15.77	5.87	2.68**	37.2	11.96	3.11**
Age	–.16	.09	–1.64	.136	.15	.93	.45	.27	1.66
Lives with husband only†	–.23	.44	–.52	–.88	.70	–1.26	4.44	1.30	3.39**
Income	–4.06	2.62	–.015	–7.39	4.08	–1.80	2.88	7.62	.37
Self-care behavior	.08	.05	1.69	–.06	.05	–1.31	–.04	.10	–.39
PP self-efficacy	–.05	.08	–.66	.00	.08	.05	.01	.17	.03
PP self-care knowledge	.02	.08	.25	.06	.08	.80	.37	.18	2.03*
Experimental group†	1.45	.72	1.98*	1.55	.89	1.74	1.62	1.74	.93
PP 2 weeks†	.47	.83	.56	.67	.71	.94	12.76	1.55	8.20***
PP 6 weeks†	–1.66	1.52	–1.08	–.51	1.46	–.35	24.54	3.12	7.84***
PP 2 weeks × experimental†	–.99	1.94	–.50	–.60	1.84	–.32	4.74	3.94	1.20
PP 6 weeks × experimental†	–6.17	1.81	–3.39*	–2.29	1.71	–1.33	13.72	3.67	3.73***

† References were living status (lived with husband and other family members), group (control), PP weeks (baseline), PP weeks×group (same week×control).

* *p*<.05,

** *p*<.01,

*** *p*<.001.

PP: Postpartum

## Supplementary materials

Further details on supplementary materials are presented online (available at https://doi.org/10.4069/kjwhn.2021.09.08).

## References

[1] Kim Y, Dee V (2017). Self-care for health in rural Hispanic women at risk for postpartum depression. Matern Child Health J.

[2] Dunning M, Seymour M, Cooklin A, Giallo R (2013). Wide awake parenting: study protocol for a randomised controlled trial of a parenting program for the management of post-partum fatigue. BMC Public Health.

[3] Giallo R, Cooklin A, Dunning M, Seymour M (2014). The efficacy of an intervention for the management of postpartum fatigue. J Obstet Gynecol Neonatal Nurs.

[4] Shafaie FS, Mirghafourvand M, Bagherinia M (2017). The association between maternal self-confidence and functional status in primiparous women during postpartum period, 2015-2016. Int J Women’s Health Reprod Sci.

[5] Barbosa EM, Sousa AA, Vasconcelos MG, Carvalho RE, Oria MO, Rodrigues DP (2016). Educational technologies to encourage (self) care in postpartum women. Rev Bras Enferm.

[6] Schaar GL, Hall M (2013). A nurse-led initiative to improve obstetricians’ screening for postpartum depression. Nurs Womens Health.

[7] Bagherinia M, Mirghafourvand M, Shafaie FS (2017). The effect of educational package on functional status and maternal self-confidence of primiparous women in postpartum period: a randomized controlled clinical trial. J Matern Fetal Neonatal Med.

[8] National Institute of Population Research and Training (NIPORT), Mitra and Associates, and ICF International (2016). Bangladesh demographic and health survey 2014.

[9] http://bbs.portal.gov.bd/sites/default/files/files/bbs.portal.gov.bd/page/4c7eb0f0_e780_4686_b546_b4fa0a8889a5/HMSS.pdf.

[10] Kamiya Y, Yoshimura Y, Islam MT (2013). An impact evaluation of the safe motherhood promotion project in Bangladesh: evidence from Japanese aid-funded technical cooperation. Soc Sci Med.

[11] Barnes J, Stuart J, Allen E, Petrou S, Sturgess J, Barlow J (2017). Randomized controlled trial and economic evaluation of nurse-led group support for young mothers during pregnancy and the first year postpartum versus usual care. Trials.

[12] Jolly SP, Rahman M, Afsana K, Yunus FM, Chowdhury AM (2016). Evaluation of maternal health service indicators in urban slum of Bangladesh. PLoS One.

[13] Birdsall KA (2014). Quiet revolution: strengthening the routine health information system in Bangladesh.

[14] http://www.mobilemamaalliance.org/sites/default/files/MAMA%20Bangladesh%20Formative%20Research%20Report.pdf.

[15] Des Jarlais DC, Lyles C, Crepaz N, TREND Group (2004). Improving the reporting quality of nonrandomized evaluations of behavioral and public health interventions: the TREND statement. Am J Public Health.

[16] Bandura A (1986). Social foundations of thought & action: a social cognitive theory.

[17] Jeon M, Hwang Y (2013). Effects of an educational program on pre-and postpartum self-care knowledge, efficacy of self-care, and interest in health of marriage immigrant women in South Korea. J Converg Inf Technol.

[18] Leahy-Warren P, McCarthy G (2011). Maternal parental self-efficacy in the postpartum period. Midwifery.

[19] Milligan RA, Parks PL, Kitzman H, Lenz ER (1997). Measuring women’s fatigue during the postpartum period. J Nurs Meas.

[20] Cox JL, Holden JM, Sagovsky R (1987). Detection of postnatal depression. Development of the 10-item Edinburgh Postnatal Depression Scale. Br J Psychiatry.

[21] Williams A, Sarker M, Ferdous ST (2018). Cultural attitudes toward postpartum depression in Dhaka, Bangladesh. Med Anthropol.

[22] Khatun F, Lee TW, Rani E, Biswas G, Raha P, Kim S (2018). The relationship among fatigue, depressive mood, self-care agency, and self-care action of first-time mothers in Bangladesh. Korean J Women Health Nurs.

[23] Barkin JL, Wisner KL (2013). The role of maternal self-care in new motherhood. Midwifery.

[24] Denyes MJ (1990). Denyes self-care instrument.

[25] Shin HS, Kim SH, Kwon SH (2000). Effects of education on primiparas’ postpartal care. Korean J Women Health Nurs.

[26] Park MK (2003). Effects of postpartum care program for primiparous women and care-givers on the knowledge and behavior of postpartum care and postpartum recovery in primiparous women [doctoral dissertation].

[27] Molenda M (2003). In search of the elusive ADDIE model. Perf Improv.

[28] Bandura A, Ramachandran VS (1994). Encyclopedia of human behavior, Vol. 4.

[29] Ho SM, Heh SS, Jevitt CM, Huang LH, Fu YY, Wang LL (2009). Effectiveness of a discharge education program in reducing the severity of postpartum depression: a randomized controlled evaluation study. Patient Educ Couns.

[30] Phipps MG, Raker CA, Ware CF, Zlotnick C (2013). Randomized controlled trial to prevent postpartum depression in adolescent mothers. Am J Obstet Gynecol.

[31] Gausia K, Fisher C, Algin S, Oosthuizen J (2007). Validation of the Bangla version of the Edinburgh Postnatal Depression Scale for a Bangladeshi sample. J Reprod Infant Psychol.

[32] Oleiwi SS, Ali RM (2010). Effectiveness of instruction-oriented intervention for primipara women upon episiotomy and self-perineal care at ibn Al-Baladi Hospital. Iraqi National J Nurs Sci.

