# Supplementary material for: Does a nurse-led postpartum self-care program for first-time mothers in Bangladesh improve postpartum fatigue, depressive mood, and maternal functioning?: a non-synchronized quasi-experimental study
Source: Korean J Women Health Nurs. 2021 Sep 30;27(3):196–208. doi: 10.4069/kjwhn.2021.09.08 (PMC9328591; doi:10.4069/kjwhn.2021.09.08)
Supplement: Supplementary file 1 [file kjwhn-2021-09-08-suppl.pdf]

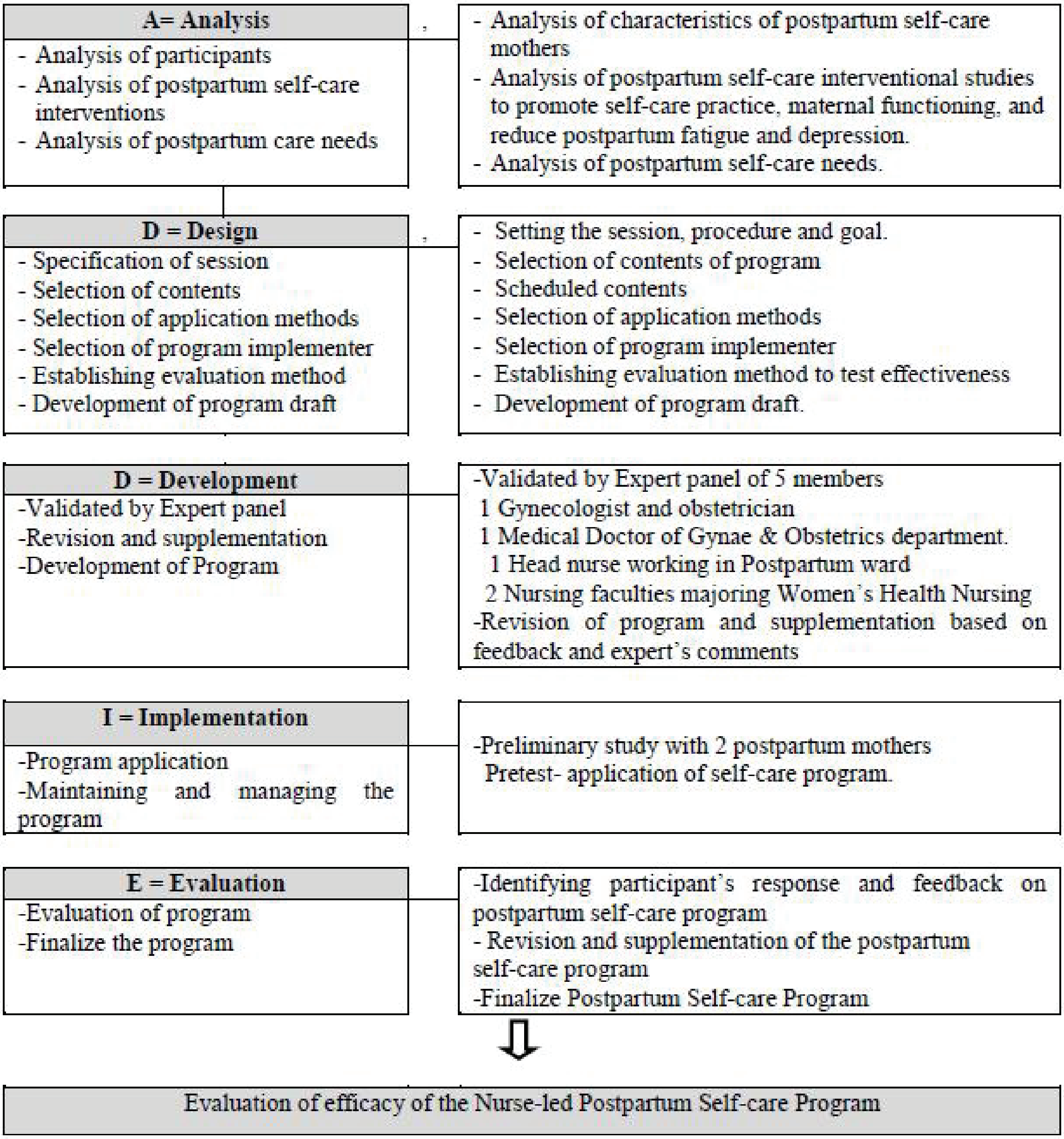

Supplementary Figure 1. Development of the Nurse-led Postpartum Self-care program according to the ADDIE process
